# Streamlining Human–Robot Interaction: Integrating LLM-Based Planning into Modular Robotic Frameworks

**DOI:** 10.3390/s26061978

**Published:** 2026-03-21

**Authors:** MinHyuk Kim, JooHee Park, Kwanyong Park, Yong-Ju Lee, Sanghun Jeon

**Affiliations:** 1 Electronics and Telecommunications Research Institute, Daejeon 34129, Republic of Korea; mhkim62@etri.re.kr (M.K.); yongju@etri.re.kr (Y.-J.L.); 2 Department of Mechanical Engineering, Korea University, Anam-ro 145, Seongbuk-gu, Seoul 02841, Republic of Korea; annaqkr@korea.ac.kr

**Keywords:** embodied AI, modular robotics, large language model, human–robot interaction, task execution

## Abstract

Embodied artificial intelligence (AI), which integrates AI and robotics, has made significant progress, particularly in human–robot interaction, task-assisting robots, and the integration of multimodal AI models. Experimental studies have demonstrated strong performance in complex tasks, such as providing human assistance, performing household chores, and object manipulation through pick-and-place operations. However, despite these impressive capabilities, real-world applicability remains limited. While tasks such as household chores and object manipulation offer significant practical utility, users often struggle to provide effective instructions, and execution remains prohibitively slow for real-world deployment. This study introduces an approach to enhance usability through spoken human instructions and reduce operation time by streamlining intermediate steps through our Module Handler. The proposed approach leverages a large language model to extract information from spoken human instructions accurately. Through experiments, we validated the accuracy of our approach and confirmed speed improvements compared with related studies. Our experiments evaluated system accuracy in extracting relevant information from spoken human instruction, achieving an object identification accuracy rate of approximately 92.47%. In addition, our method reduced task completion times by an average of 33 s across four different experimental environments compared with existing modular robotics systems. This time reduction is significant for enhancing robotic task execution efficiency.

## 1. Introduction

Traditional robotics relies heavily on pre-programmed instructions and performs effectively in structured and predictable environments [1,2]. However, this approach poses several challenges when robots are required to execute complex tasks in dynamic real-world settings, such as handling unprogrammed objects, adapting to unfamiliar scenarios, and responding to human interactions [3]. For instance, a robot governed by rigid instructions often fail to handle novel objects omitted from their initial programming. Such systems typically lack the situational flexibility required for dynamic environments, thereby hindering their overall adaptability in unpredictable real-world scenarios [1,2,3]. To address these limitations, numerous research efforts [4,5] have attempted to integrate perception, cognition, and action within a physical entity, enabling robots to interact with their environments. This research has led to the concept of embodied artificial intelligence (AI), which leverages advanced AI techniques to allow robots to adapt to diverse environments and perform complex tasks [5]. Examples of such methods include utilizing sensors such as LiDAR, cameras, and microphones for object detection and decision-making [6,7]; applying simulation-trained models to real-world environments [8,9]; and exploring navigation and path planning for agent movement [10].

The recent development of large language models (LLMs) has propelled this field in various directions. Researchers have leveraged LLMs such as ChatGPT-4o mini [11] (OpenAI, San Francisco, CA, USA) to generate robot control codes [12,13], facilitating the control of multiple robotic platforms [12,14,15,16], including robotic arms, drones, and language-assisted home robots, without extensive programming. Additionally, humanoid robots powered by models such as GPT-4 [17] have been developed, which can perform human-like actions and understand natural language commands without prior programming or training. Furthermore, robot navigation systems utilizing zero-shot object navigation technology and LLM frameworks [18] have been created to identify target objects through conversational interactions, enhancing the ability of robots to navigate and locate objects without prior knowledge.

Despite these advancements, current embodied AI research often faces challenges when transitioning from experimental settings to practical applications. Although several studies have considered user convenience, most task commands and intermediate steps are executed within command-line interface environments [12,14,15,16], which require expert knowledge for operation. This reliance on technical interfaces makes it difficult for untrained end users to interact effectively with robotic systems. Moreover, as noted in several comprehensive surveys [5,19,20], while research has demonstrated impressive capabilities, the speed and efficiency of task execution remain a significant challenge for the practical, real-life application of robotic agents. Finally, conducting studies primarily in simulation environments introduces a sim-to-real gap [21,22]: a discrepancy between the observed performance in simulations and that in real-world scenarios.

To address these limitations, we propose an integrated approach that enhances both user convenience and task execution speed. We improve the user convenience by enabling auditory interaction between the user and robotic agent. By combining a speech-to-text model with an LLM that is pretrained on open-vocabulary datasets, the system can comprehend natural language commands, allowing users to issue instructions in a more intuitive and accessible manner. To reduce interruptions during task planning in the intermediate steps, we implement a streamlined module. The handler adaptively maps tasks from the initial command, significantly reducing the overall time required for the agent to perform tasks. This enhancement increases the efficiency and makes the system more suitable for real-world applications. Furthermore, our approach avoids additional sim-to-real transfer steps, as all experiments and validations were conducted directly in real-world environments. By avoiding simulations, our models and systems are immediately applicable, without additional adaptation steps.

The contributions of this paper are threefold:

LLM-based Auto-Planner Module: We propose an LLM-based Auto-Planner Module that enhances the ability of a robot to comprehend ambiguous or complex commands. By leveraging an LLM with custom prompts, this module effectively interprets spoken language and identifies key objects. Once the auto-planner extracts the relevant objects and tasks, the Module Handler assigns tasks to the appropriate modules, optimizing the system workflow. This auto-planner indirectly contributes to system efficiency by improving the command comprehension and task allocation.

Module Handler: We introduce a Module Handler that coordinates and manages the execution of various modules within the system. The Module Handler significantly reduces the overall execution time by eliminating the need for manual command inputs for module activation. This structured approach ensures smooth coordination between components, integrating the LLM-based auto-planner with other modules seamlessly, enhancing system efficiency, adaptability, and ease of maintenance.

Modular Construction: We structure the robot functions into a modular format, including a voice recognition module and an LLM-based auto-planner module, thereby allowing seamless adaptability to other modular robotics frameworks.

Our integrated approach addresses the technical challenges associated with embodied AI while also prioritizing user experience, which is a critical factor for the adoption of robotic systems in daily life. Experimental results validated the ability of the LLM-based auto-planner to interpret ambiguous or complex voice commands accurately, achieving a success rate of approximately 92.47% in object and task identification. Additionally, our system demonstrated improved execution times in real-world environments compared with existing studies.

## 2. Related Works

### 2.1. Embodied AI

Embodied AI is an emerging field that integrates physical agents with advanced AI capabilities, enabling them to perceive, learn, and interact with the real world in human-like manners [23]. Recent embodied AI research has focused on combining deep learning, reinforcement learning [1], and sensor technologies to create robots and agents that can adapt to complex, unstructured environments [6,24]. Researchers are developing systems that leverage vision, touch, and auditory inputs to improve perception and decision-making processes [10,25]. In addition, there is an increasing emphasis on incorporating natural language processing and LLMs to enhance human–robot interactions, allowing robots to understand and respond to spoken or written commands more effectively [26,27].

Despite these advances, embodied AI still faces significant challenges. A primary issue is the sim-to-real gap, in which models that are trained in simulated environments often underperform in real-world settings owing to differences in dynamics and sensory inputs [5,21,22]. Bridging this gap necessitates extensive real-world data collection and adaptation techniques, which can be both time consuming and resource intensive. Furthermore, deploying complex AI models for physical robots introduces computational constraints that affect the real-time performance and scalability [6]. Safety and reliability are also critical concerns, particularly for robots operating autonomously in unpredictable environments [28,29]. Ethical considerations, such as ensuring transparency and preventing bias in decision-making, present additional challenges [30,31]. Addressing these limitations is essential for enabling the practical application of embodied AI in everyday scenarios.

### 2.2. Navigation Systems

Embodied AI has significantly advanced robotic navigation systems, particularly in enabling robots to navigate paths toward objects autonomously in complex, unstructured environments [14,15,16,32]. Traditional navigation methods often rely on predefined maps and specific object recognition algorithms, which limits robot adaptability in dynamic settings [33]. However, recent advancements focus on integrating sensing, perception, and action capabilities to allow robots to develop a semantic understanding of their surroundings.

Building upon this trend, recent approaches have started to explore the use of memory-driven, language-informed reasoning mechanisms to enhance semantic navigation further. For instance, NaviLLM [34] introduces a novel paradigm that embeds planning and decision-making processes directly within an LLM. Rather than relying on explicit geometric maps, NaviLLM dynamically constructs a multimodal context from textual instructions, action history, and panoramic visual observations. It utilizes a schema-based instruction mechanism to generate the next navigational action as a token, effectively replacing traditional planners with a generative reasoning engine. Similarly, CLIP-Fields [35] constructs an implicit spatial memory using weak supervision from large-scale web models such as CLIP [36] and Multilingual E5 [37]. This architecture enables robots to perform tasks such as semantic segmentation and object navigation in real-world 3D environments with minimal human intervention. These models mark a shift from traditional, geometry-based navigation to a semantics-driven paradigm, which involves integrating multimodal inputs to support contextual understanding and reasoning.

In contrast to these approaches, our framework integrates LLM-based semantic planning with zero-shot spatial mapping for direct real-world deployment. While NaviLLM is primarily simulation-focused, our semantic planning translates complex linguistic intent into actionable plans for physical environments. Additionally, unlike CLIP-Fields, which requires map-specific training, our method utilizes an ensemble of pre-trained multimodal models to construct a VoxelMap without additional fine-tuning, allowing for immediate autonomous interaction in unfamiliar spaces.

### 2.3. Object Manipulation Systems

Object manipulation and pick-and-place tasks are essential capabilities for robots operating in real-world environments. With recent advancements in embodied AI, robots now integrate sensing, planning, and execution far more tightly, enabling effective interaction with objects even in unstructured settings.

AnySkin [38] represents the latest push toward skin sensing—equipping grippers with plug-and-play magnetic tactile skins that provide fine-grained, high-sensitivity contact feedback that is well suited to everyday use. By correcting signal inconsistencies between sensor instances, AnySkin supports the zero-shot transfer of visuotactile policies when a sensor is swapped, incurring only a 13% performance reduction (versus 43% in prior work). This robustness suggests strong potential for scalable, reliable manipulation in dynamic, real-world settings. EgoZero [23] tackles the data bottleneck from another angle: it collects realistic, egocentric demonstrations with smart glasses and learns policies that simply mimic human pick-and-place motions. A morphology-agnostic state–action representation allows the resulting model to transfer directly to a robot without additional robot-specific data. The OK-Robot [39] system, which is an open knowledge-based robotic system, integrates various pretrained vision-language models (VLMs), such as CLIP, LangSAM [40], AnyGrasp [41], and OWL-ViT [42], with navigation and grasping modules for open-vocabulary object manipulation. By leveraging these pretrained models, OK-Robot eliminates the need for task-specific training on environmental data, images, or language instructions. Collectively, these systems demonstrate a shift from controlled laboratory manipulation to approaches that are designed for dynamic, real-world scenarios.

## 3. Materials and Methods

**Figure 1:**
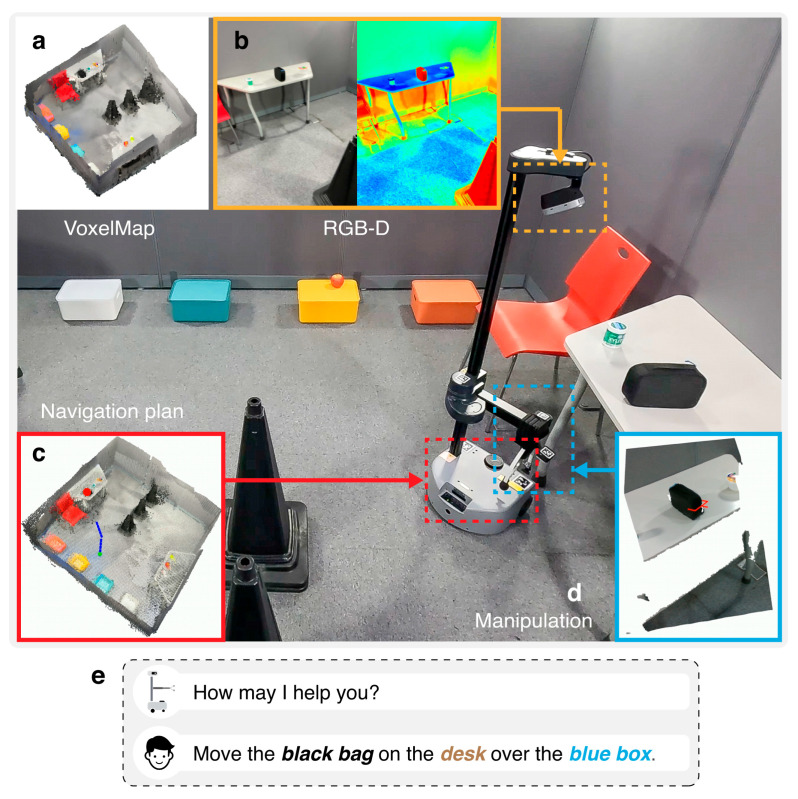
Overview of the robotic system components. (**a**) A VoxelMap is constructed for navigation using (**b**) captured RGB and depth images. (**c**) Voice commands from the user guide the system. (**d**) A navigation plan is generated for efficient movement. (**e**) Object manipulation is performed based on the plan.

Our method is designed to enhance the cognitive capabilities of modular robotic frameworks. By leveraging the modular structure, the proposed approach can be applied across various existing robotic systems that utilize independent modules for tasks such as perception, navigation, and manipulation. Among these frameworks, we selected the OK-Robot architecture owing to its comprehensive integration of multiple VLMs and adaptability to diverse robotics applications. To extend the capabilities of the original OK-Robot architecture, we retain the foundational modules that are responsible for ***Scanning*** (Figure 1a,b), which acquire and process RGB-D data to construct a spatial understanding of the environment; ***Navigation*** (Figure 1d), which plans and executes optimal movement paths; and ***Manipulation*** (Figure 1e), which handles object detection, grasp planning, and placement actions. In addition to these core components, we introduce the novel ***Interaction*** module (Figure 1c), which is specifically designed to sense spoken commands and scene images, transmit this data to the Central Server, and subsequently receive and execute coordinated action commands.

**Figure 2:**
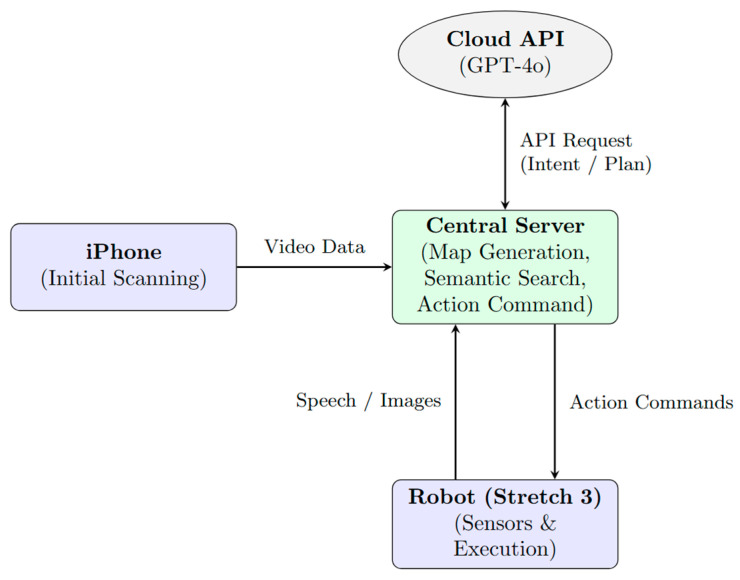
System architecture and data flow across hardware components.

As illustrated in the system architecture (Figure 2), this distributed setup optimizes processing by separating initialization, reasoning, and execution. During the initial initialization phase, an iPhone 12 Pro (Apple, Cupertino, CA, USA) captures the environment and relays the data to the server for map generation. During operation, the Central Server performs semantic reasoning through the Cloud API (GPT-4o mini) and transmits structured task plans back to the robot. This allows the system to perform robust, context-aware actions in complex environments while maintaining efficient, low-latency control on the robotic platform.

**Figure 3:**
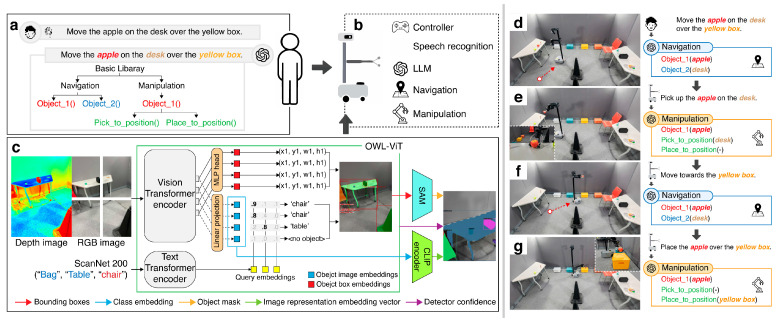
Overview of the proposed system architecture. (**a**) The auto-planner interprets spoken language and context to extract objects and actions, generating a structured task plan. (**b**) The robot executes the plan by autonomously navigating and manipulating objects. (**c**) Architecture of the object-centric semantic memory, showing how images are processed to construct the VoxelMap. (**d**–**g**) Real-world execution demonstrating navigation, object identification, and manipulation based on the planned actions.

During the Scanning phase, the environment is captured using an RGB-D camera and the data are converted into an ***object-centric semantic memory***, which is referred to as the VoxelMap [39], following prior research. A key aspect of our approach is the design of the Interaction module to utilize the VoxelMap effectively, ensuring seamless integration and optimal system performance. Furthermore, by leveraging the modular architecture of OK-Robot, all functionalities, including the newly introduced Interaction module, are implemented in a modular format. The modular design of the system (Figure 3b) facilitates scalability and maintainability by allowing individual components to be developed, tested, and updated independently. This architectural flexibility not only simplifies system integration and debugging but also enables seamless incorporation of future functional enhancements and hardware or software extensions.

### 3.1. Scanning

The Scanning phase (Figure 3c) is responsible for constructing a detailed 3D representation of the environment surrounding the robot by processing RGB-D sensor data. This map serves as the foundation for subsequent modules by capturing spatial features, identifying object locations, and enabling semantic understanding of the scene. This is accomplished using the Record3D application on an iPhone, which captures RGB-D images of the surroundings. These images are converted into voxel maps, with depth data from the RGB-D images re-projected to create a coordinate representation vector that captures the spatial layout of the environment. Subsequently, the visual feature vector $v_{i}\in R^{768}$ is transformed into a lower-dimensional representation ${\hat{v}}_{i}\in R^{512}$ via a linear projection layer $\phi \left(\cdot \right)$, such that ${\hat{v}}_{i}=\phi \left(v_{i}\right)$. To ensure robust semantic matching, L2 normalization is applied to both the projected voxel feature ${\hat{v}}_{i}$ and the text query embedding $t\in R^{512}$. The similarity score S for identifying target objects is then calculated using cosine similarity as follows:

$$S=\frac{{\hat{v}}_{i}\cdot t}{|{\hat{v}}_{i}{|}_{2}|t{|}_{2}}$$

**Figure 4:**
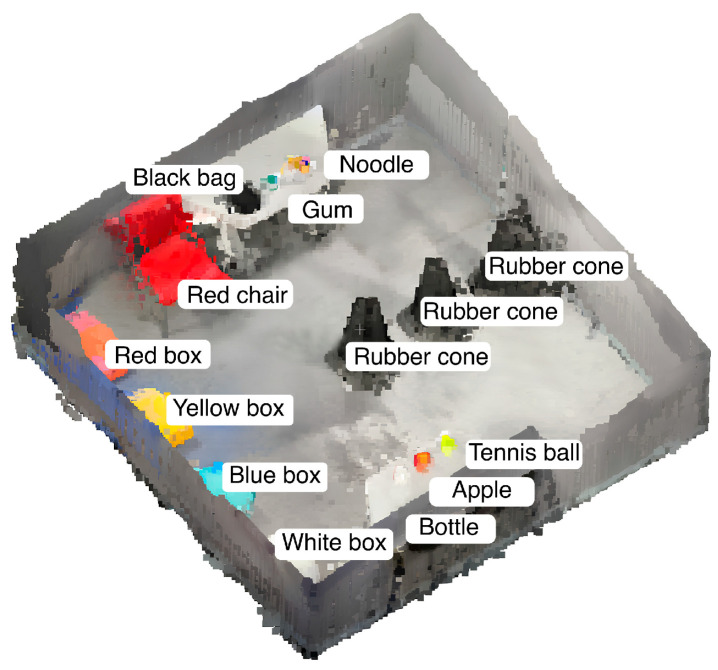
VoxelMap representation of the environment, with labeled objects and obstacles. Items such as a black bag, tennis ball, red chair, and various boxes are annotated with their respective labels.

This unified voxel-based representation encodes both semantic attributes and precise spatial location information within a single high-dimensional vector structure. VoxelMap provides the robot with a complete 3D representation of its environment, serving as the foundation for subsequent phases and enabling precise navigation and interaction within the space, as illustrated in Figure 4.

### 3.2. Navigation

Building on the VoxelMap generated during the Scanning phase, the Navigation phase guides the robot through the environment. When a user inputs a query, such as requesting an object to be moved or specifying its destination, the model converts the query into a representation vector similar to that used in the Scanning phase. The system then searches for the most similar voxels by comparing the query representation with each voxel in the VoxelMap. The system can accurately identify the target by calculating the similarity between these representations.

Additionally, the integration of a heuristic-enhanced A* algorithm enables the robot to compute the optimal path and avoid obstacles while navigating the environment. This combination ensures that the robot can move seamlessly through complex environments, reaching designated targets with precision.

### 3.3. Manipulation

The Manipulation phase consists of two primary components: grasp and place. (1) Grasping: Upon reaching the object location, the robot uses an RGB-D camera to capture its 3D position, converting the depth image into a point cloud. The pretrained AnyGrasp model generates several collision-free grasp poses based on the RGB images and point cloud data. These proposed grasps are then filtered using LangSAM, which is a language-based segmentation model that identifies the target object within the scene. The selection of the optimal grasp pose $g^{*}$ is performed through a multi-stage refinement pipeline. From the raw candidates $G=\{\left(g_{i},s_{i}\right){\}}_{i=1}^{N}$ generated by the detection network, the system first applies Non-Maximum Suppression (NMS) to eliminate redundancy. The remaining candidates are then subjected to a two-fold feasibility filter: (1) Reachability, each candidate is verified for a valid Inverse Kinematics (IK) solution within the robot’s kinematic workspace, and (2) Collision, by cross-referencing the gripper geometry with the 3D VoxelMap. The final grasp pose is determined by: $g^{*}=\arg\underset{g_{i}\in G^{\prime }}{\max}\;s_{i}$ where $G^{’}$ represents the set of filtered, executable candidates. This selection process ensures that the robot prioritizes the most stable grasp that is also kinematically reachable and collision-free.

The robot approaches the object with controlled, gradual movements and executes a secure grasp to ensure a reliable hold. (2) Placing: After securing the object, the robot navigates to the designated location using the previously described navigation methods. The head-mounted camera of the robot captures a point cloud of the destination, which is segmented and aligned based on language inputs via LangSAM. The segmented point cloud is processed to calculate an appropriate drop height, with a buffer added to account for obstacles. The robot positions the gripper above the drop point and releases the object, ensuring that it is placed securely without collision.

### 3.4. Interaction

The Interaction phase (Figure 3a) serves as a core component of the proposed system, comprising multiple integrated modules that are designed to interpret user-issued natural language commands, extract actionable intent, and translate them into structured task plans. This phase enables the robot to understand complex instructions and coordinate subsequent behaviors that are required for autonomous task execution.

*Speech Recognition Module*: The process begins with OpenAI’s Whisper large-v3-turbo [43] (OpenAI, San Francisco, CA, USA) for speech recognition, selected for its superior accuracy even in challenging noisy environments, after evaluating other noise-robust models [25,44]. Whisper converts spoken language into text with high precision, forming the foundation for natural language interactions between robots and users.

Auto-Planner Module: Once speech is converted into text, the input is processed using GPT-4o-mini [17], which serves as the core of the auto-planner. GPT-4o-mini, which is optimized for rapid inference, is guided by custom scripts to identify objects, destinations, and required tasks automatically. For further details on specific prompts, see Appendix A.1. As described in the evaluation section, this module was tested on 1900 generated example tasks to extract the correct targets, achieving a high accuracy rate of 92.47%, thereby demonstrating its effectiveness for real-world task comprehension and execution.

Module Handler: Based on outputs from the auto-planner, the Module Handler efficiently directs each phase of the robot operation. It assigns the extracted information to the appropriate modules for task execution, reducing redundant steps and improving the overall efficiency.

In summary, the Interaction phase—comprising the Speech Recognition Module, Auto-Planner Module, and Module Handler—represents a substantial improvement over the capabilities of the original system.

## 4. Experiments

Minimizing the execution time is essential to enhance the practical utility of robotic systems in real-world scenarios. To assess the effectiveness of our proposed method, we conducted experiments comparing the inference time of the enhanced system with that of the original OK-Robot.

**Figure 5:**
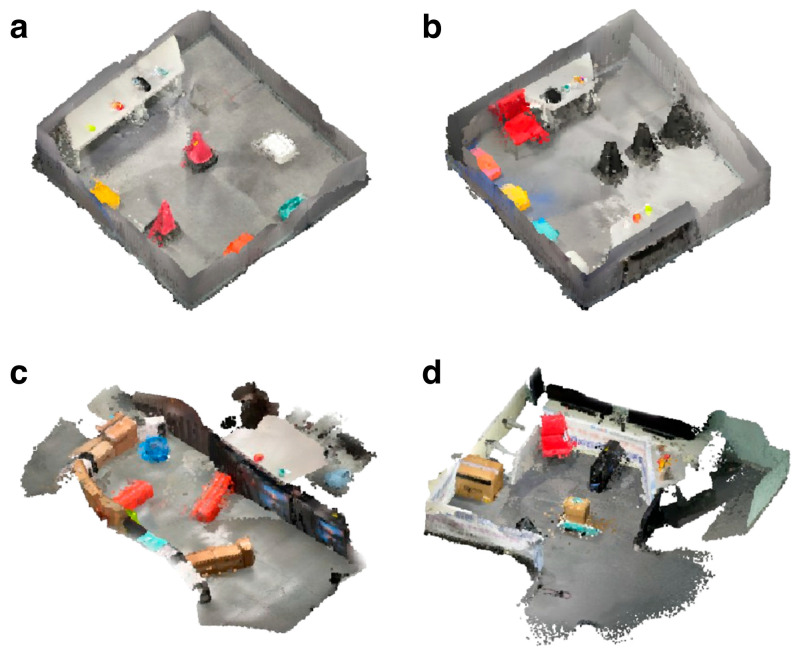
VoxelMap representations of the experimental environments used to evaluate the task execution time. (**a**,**b**) depict **closed-space** environments, whereas (**c**,**d**) illustrate **open-space** environments.

To evaluate the task execution performance of the system under varying spatial constraints rigorously, we designed four distinct experimental environments, as illustrated in Figure 5. These environments are categorized into two types: (a) and (b) represent compact, square-shaped closed spaces intended for testing short-range navigation and manipulation tasks, whereas (c) and (d) depict wide, open-space settings that are designed to assess the ability of the system to handle long-range task execution, spatial adaptability, and dynamic planning across extended areas. The square space required shorter execution times but posed challenges in obstacle avoidance, whereas the wider space allowed us to evaluate the capacity of the system to handle more complex tasks over extended durations.

In each environment, we performed two consecutive pick-and-place tasks, comparing the execution time of the original method with that of the enhanced approach. In the original method, each command needed to be manually typed for every module function. In contrast, our method requires only a single speech command to integrate the entire process, significantly reducing user input and improving operational efficiency.

### 4.1. Real-World Environment

To evaluate the performance of our system comprehensively, we designed four experimental environments with varying complexities and layouts to test the ability of the robot to navigate obstacles, perform pick-and-place tasks, and manage different spatial constraints.

Closed space A: In this scenario (Figure 5a), the robot operates in a square-shaped environment with two red rubber cones positioned as obstacles. A desk along the wall contains various objects, including tennis balls, apples, black bags, noodles, and gums. Four boxes—yellow, red, blue, and white—are designated as placement destinations. The task of the robot is to pick up a tennis ball and place it in the white box, followed by picking up gum and placing it in the red box. This setup tests the capacity of the robot to perform tasks in a confined space with nearby obstacles.

Closed space B: Similar in structure to closed space A, this environment (Figure 5b) introduces increased complexity with two desks placed against the walls and three black rubber cones as obstacles. The same set of objects from Scenario A is used. In this case, the task of the robot is to pick up an apple and place it in the yellow box, followed by picking up a black bag and placing it in the blue box. This scenario tests the navigation and accuracy of the robot in a more cluttered environment.

Open space C: In this open environment (Figure 5c), multiple box-shaped obstacles are stacked to create routes for the robot to navigate. Two buckets—blue and green—are designated as placement destinations. The same objects used in Scenario A are included. However, the pick-and-place tasks mirror those in Scenario A, with the robot first placing the tennis ball in the blue bucket, followed by placing the gum in the green bucket. This environment is designed to assess the efficiency of the robot in long-range navigation and adaptability within a structured, obstacle-dense space.

Open space D: The final environment (Figure 5d) features an open space in which box obstacles are arranged centrally, creating multiple possible routes for the robot to navigate. In this scenario, the robot performs pick-and-place tasks using objects such as pink bottles, apples, peaches, and small boxes. The tasks involve the robot first picking up an apple and placing it in a large box, followed by picking up a pink bottle and placing it on a red chair. This setup is designed to assess whether our method remains effective in scenarios in which the robot must determine optimal paths from multiple options and navigate through more complex routes.

### 4.2. Experimental Setup and Hardware

**Figure 6:**
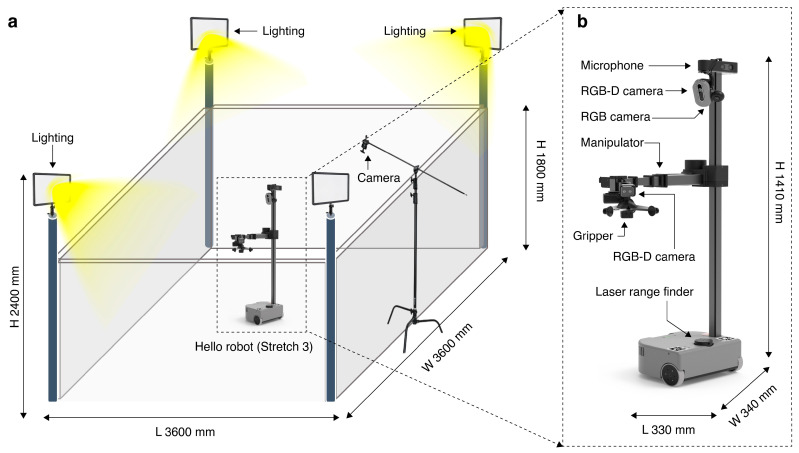
(**a**) Hardware environment with closed space and (**b**) hardware specifications of Stretch 3 robot.

The workspace (Figure 6a), measuring 3600 mm in length and width and 2400 mm in height, was illuminated by three strategically positioned lights. Multiple cameras were installed to capture a comprehensive view of the robot interactions, enabling precise tracking and analysis under standardized lighting conditions.

The Stretch 3 (Hello Robot, Atlanta, GA, USA) robot (Figure 6b) has a compact size of 33 × 34 × 141 cm and weighs 24.5 kg, supporting operations for 2 to 5 h, depending on the CPU load. It moves at a maximum speed of 30 cm/s and can carry up to 10 kg on its mobile base. Its Cartesian manipulator provides a lift range of 110 cm, handling loads of up to 5 kg, while the arm can manage up to 3 kg. The robot, which is equipped with an Intel RealSense D435i depth camera (Intel, Santa Clara, CA, USA) and a 340-degree laser rangefinder, excels in navigation and object detection. The integrated Intel i5-1240P processor with 16 GB RAM and a 512 GB SSD ensures high performance. Its gripper opens up to 15 cm, supporting objects up to 2 kg, while the DexWrist 3 can handle up to 2.5 kg with versatile wrist motions.

However, owing to compatibility requirements with our base framework, OK-Robot, which was initially developed for the Stretch 2 robot, we had to modify the software environment. Specifically, the OK-Robot software infrastructure relies on earlier versions of Python 3.10 (Python Software Foundation, Wilmington, DE, USA) and the robot operating system (ROS) Noetic (Open Robotics, Mountain View, CA, USA), which are not natively supported by the updated hardware and software stack of the Stretch 3 robot. Consequently, we downgraded the software of the Stretch 3 robot and adapted it to function with older versions of Python and the ROS. This modification enabled the full integration of the hardware components of Stretch 3 with the OK-Robot framework, ensuring compatibility and functionality throughout the experimental process.

### 4.3. Task Flow and Module Processing

**Figure 7:**
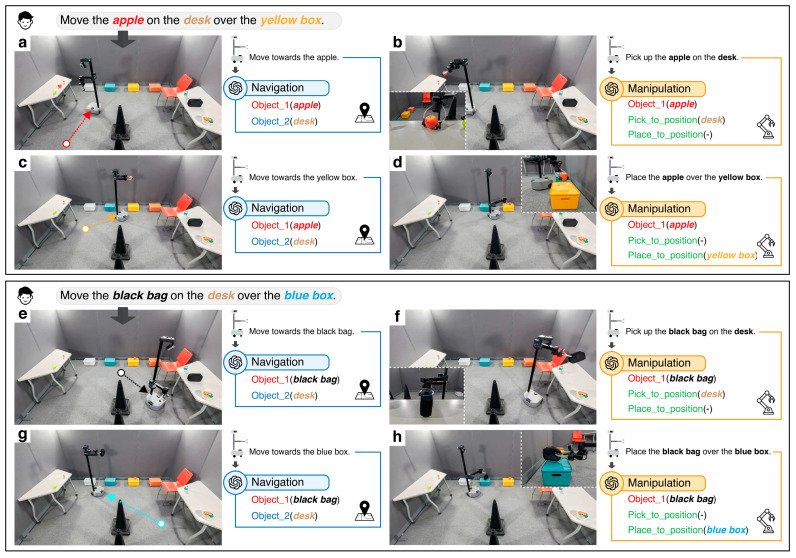
Task flow and module processing for two consecutive pick-and-place tasks. (**a**) Navigation for the first task—object search and route planning; (**b**) Manipulation for the first task—object detection, grasp pose identification, and execution; (**c**) Navigation for the first task—route planning toward the placement destination; (**d**) Manipulation for the first task—placement execution; (**e**) Navigation for the second task—object search and route planning; (**f**) Manipulation for the second task—object detection and grasp pose identification; (**g**) Navigation for the second task—route planning toward the placement destination; (**h**) Manipulation for the second task—placement execution.

As illustrated in Figure 7a, the task flow was divided into two consecutive pick-and-place tasks to ensure the robustness of our model by testing its adaptability across diverse conditions. Specifically, during these consecutive tasks, the starting points of the robot varied as it repositioned itself after each object placement, challenging the ability of the model to recalibrate navigation paths dynamically.

The system operates based on voice commands. For example, a command such as “Please move the apple from the desk to the yellow box” initiates the task. Upon receiving the command, our LLM-based Auto-Planner Module processes the input, extracting key information such as the object to be moved and its destination. This information is then passed to the Module Handler, which assigns the relevant details to the appropriate modules for execution.

In this scenario, the robot performed sequential tasks while adapting to positional variations. Each task was executed using two primary modules: Navigation and Manipulation. The Navigation module, as illustrated in stages in Figure 7a,c,e,g, first searches the VoxelMap for the target object, identifying the voxel that best matches the input object. After locating the object, the module calculates the optimal route to the voxel, ensuring efficient navigation toward the target. This process, which is detailed in Section 3.2, demonstrates the effectiveness of the model for both object search and navigation. For object placement, the same navigation process is applied, but with the search query modified to locate the target voxel for object placement instead of the object itself. This consistent approach ensures precision in both object grasping and placement tasks, underscoring the adaptability of the model in dynamic real-world environments.

The Manipulation module, as shown in stages in Figure 7b,d,f,h, handles the precise actions required for grasping and placing objects. Once the target object is reached, the Manipulation module activates the D435i depth camera to facilitate accurate object handling. To determine the exact position of an object, the system uses AnyGrasp to generate candidate grasping poses, predicting multiple potential grasps based on the geometry and position of the object. The system then selects the optimal grasping pose to ensure a secure hold onto the object.

Similarly, when the robot reaches the placement destination, the Manipulation module performs the object placement task. The D435i depth camera is used to locate the precise target position for placement, and the robot executes the action according to the workflow detailed in Section 3.3. This consistent approach across both grasping and placement tasks enables the robot to maintain high accuracy and adaptability, regardless of variations in the object size, shape, or environmental dynamics.

## 5. Results

In this section, we present a comprehensive evaluation of our adapted system, focusing on two key aspects: running-time reduction and voice-command processing accuracy. First, we compared the execution times of the original OK-Robot system with those of our method to demonstrate the improvements achieved through the integration of the Module Handler. To evaluate the performance further, we conducted five trials in each experimental environment and calculated the average execution time for each module. We also report the standard deviation and total execution time for each scenario to provide a thorough assessment of the system performance and consistency.

Next, we assessed the accuracy of our voice recognition system to ensure that the intended objects were correctly identified and processed based on voice commands. This assessment, which was conducted across 1900 sample tasks, measured the effectiveness of the system in accurately extracting target objects. These evaluations highlighted the efficiency and robustness of the proposed approach in real-world scenarios.

### 5.1. Execution Time Comparison

**Figure 8:**
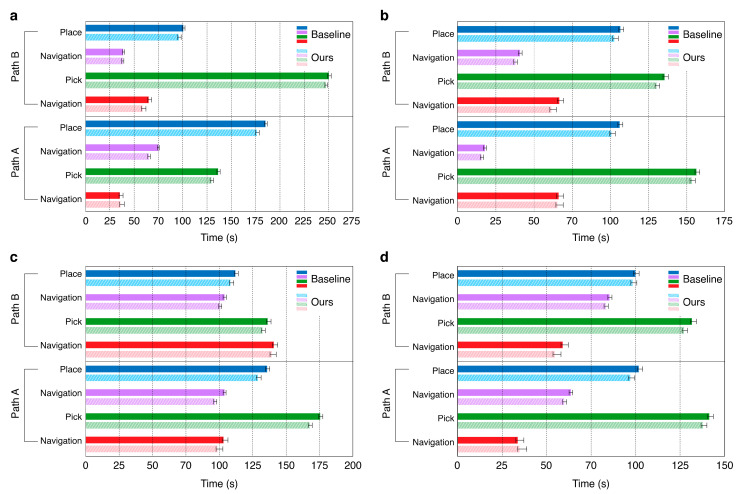
Execution time comparison across different environments (Maps a–d) shown in Figure 5. Paths A and B refer to two consecutive pick-and-place tasks performed within each map. (**a**,**b**) depict closed-space environments, whereas (**c**,**d**) illustrate open-space environments.

We compared the average total execution times of the original OK-Robot method with our proposed approach across both the square-space and open-space environments, as summarized in Figure 8 and Appendix A.2. In all experimental environments, our method consistently reduced the total execution time compared with the original OK-Robot system.

In the square-space environments (Maps a and b), the original method recorded average total times of 717 and 698 s, respectively, whereas our method reduced these times to 693 and 670 s. In the open-space environments (Maps c and d), the original method recorded average times of 1012 and 886 s, while our approach reduced these to 973 and 846 s, respectively.

The most substantial reduction in execution time was observed in Map D, where our method achieved a 40 s decrease compared with the original system. When averaging the results across five trials for each of the four maps, the cumulative time difference across all trials was 2 min and 10 s. To validate the statistical significance of these improvements, we performed a paired *t*-test across all 20 trials. The analysis revealed a highly significant improvement (*p* < 0.001), with a mean reduction of 32.8 s and a 95% confidence interval (CI) of [29.0, 36.6] s. Furthermore, a post hoc power analysis yielded a statistical power greater than 0.99 with a large effect size (Cohen’s d = 4.04), confirming that the sample size provided sufficient evidentiary weight to support our conclusions according to established reporting standards [45]

### 5.2. Voice Command Processing

To evaluate the accuracy of the system in extracting target information from voice commands, we employed a structured approach using GPT-4o mini. First, we asked GPT to generate a list of 100 objects that can be easily moved by hand and a list of 100 locations that are commonly found in home environments in which objects could be placed. Using these lists, we generated 1900 text commands instructing the system to move specific objects from one location to another. Detailed prompts and example result from this process are presented in Appendix A.3. To convert these text commands into voice commands, we used OpenAI’s Audio API, specifically tts-1 and tts-1-hd, with random selection between the two. In addition, the tone of voice was randomly varied across six different types to ensure diversity. This approach allowed us to create a robust set of voice commands, providing a comprehensive evaluation of the ability of the system to process and execute tasks based on natural language input.

Using this dataset, we evaluated the accuracy of the system in identifying the correct object. The results showed an accuracy rate of 92.47% for target object extraction, demonstrating that our system can reliably process diverse voice inputs. This high accuracy level ensures effective performance in real-world applications, even with varied user commands, thereby reinforcing its practical usability and adaptability.

## 6. Discussion

In this study, we enhanced the existing OK-Robot system by integrating the LLM-based Auto-Planner Module, voice command system, and Module Handler, significantly improving the interaction capabilities of the robot. Specifically, the voice recognition system demonstrated an accuracy of 92.47% in extracting target objects from diverse voice commands, validating its high reliability. To evaluate the system performance, we conducted experiments across four different environments, with each environment tested five times, totaling 20 trials. The results confirmed a substantial reduction in the execution time for pick-and-place tasks compared with the original system. This improvement is primarily attributed to the Module Handler, which, as opposed to traditional methods that require sequential module activations after the execution of each module, automatically orchestrates the entire sequence of modules based on the initial command. This eliminates repetitive decision-making and coordination between modules, significantly reducing the overall execution time. Quantitatively, the average execution time was reduced by 39 s and 40 s on the more demanding paths C and D, respectively, whereas the simpler paths A and B registered reductions of 24 s and 28 s, respectively. These results underscore the greater efficiency of the proposed method in complex, long-distance scenarios. Preliminary observations further suggest that spatial constraints influence the speed and accuracy of the robot, indicating performance differences between confined and open environments. A rigorous, fine-grained quantitative analysis of this relationship will be conducted in future work.

Although this study achieved meaningful results, several limitations remain to be addressed. First, the current VoxelMap is static: once it has been generated, it cannot be updated during execution. This limitation prevents the robot from adapting to moving or newly appearing objects and obstacles, reducing its flexibility in dynamic scenarios and making it difficult to perform prolonged or continuous experiments. Second, the VoxelMap construction depends entirely on depth sensing, which is susceptible to noise in low-light conditions or near reflective surfaces. These sensor limitations degrade map quality, subsequently compromising the precision of both navigation and manipulation.

These limitations sometimes slowed down the task execution. The robot required extra time to adjust when objects moved, and reflective surfaces introduced depth sensor noise that required additional processing. As a result, we completed only 20 trials (five runs in each of the four environments). Although this modest sample size is adequate for detecting general performance trends, it is insufficient for drawing statistically rigorous conclusions. Future work with real-time map updates and more robust sensors will enable larger-scale tests and provide a clearer demonstration of the performance variability.

## 7. Conclusions

This study has introduced an improved OK-Robot system incorporating the LLM-based Auto-Planner Module, voice command interface, and Module Handler to enhance task efficiency and user interaction. The study successfully demonstrated high voice recognition accuracy and improved task execution speeds; however, limitations such as the static nature of the VoxelMap and a limited number of experiments were clearly identified. These findings underscore the necessity for further quantitative research to enhance the practical applicability of the system.

Future research will focus on developing technologies for dynamically updating the VoxelMap in real time, thereby improving the flexibility and long-term task performance of the system. Through this advancement, we aim to conduct more extensive experimental evaluations to achieve statistically robust quantitative analyses. In addition, future work will quantitatively assess each module (navigation, pick, place, instruction recognition, and module handling) to provide more reliable numerical and mathematical evaluations. Ultimately, ongoing research will aim to maximize the adaptability of the robot to environments and to enhance intuitive human–robot interactions.

## Data Availability

The original contributions presented in this study are included in the article. Further inquiries can be directed to the corresponding author.

## Abbreviations

The following abbreviations are used in this manuscript:

AI	Artificial Intelligence
LiDAR	Light Detection and Ranging
LLMs	Large language models
VLMs	Vision-language models
ROS	Robot operating system
SAM	Segment Anything Model

## Appendix A

This Appendix presents the two main components.

Prompt scripts were utilized with the large language model (LLM) during the experimental phase.

Execution time metrics were collected across various simulated environments (Maps a–d), as visualized in Figure 5.

Example sentences and environmental terms were used to generate data during the validation phase.

These materials were provided to enhance the reproducibility and transparency of the research and facilitate further studies and applications based on this work.

### Appendix A.1

**Listing A1.** Prompts used with the LLM to generate datasets for validating the performance of the speech recognition module. These prompts were specifically designed to create requests related to moving household objects to different locations within the home environment, simulating realistic task scenarios for evaluation.
messages = [ { “role”: “system”, “content”: [ { “type”: “text”, “text”: “““““ You are an assistant skilled in extracting key information about objects and locations from natural language instructions. You will be provided with a sentence that tells you to pick up an object from a specific place and put it somewhere else. Your task is to extract three things: 1. The **object** (including typical color and material). 2. The **place** where the object is located. 3. The **destination** where the object should be placed. Format your response as: object with typical color and material, place, destination. Important: - If the object is uncommon or uses a brand name, replace it with a more common name. - Focus on the general type of material and typical color for common objects. Examples: Input: “Pick up the blue Samsung phone from the kitchen table and place it on the office desk.” Output: blue Samsung phone, kitchen table, office desk Input: “Grab the leather wallet from the living room couch and move it to the bedroom dresser.” Output: leather wallet, living room couch, bedroom dresser Input: “Take the red Nike sneakers from the hallway closet and put them in the shoe rack by the front door.” Output: red Nike sneakers, hallway closet, shoe rack by the front door “““ } ] } ]

### Appendix A.2

**Table A1:** Execution time comparison across different environments (Maps a–d) shown in Figure 5. A.T. denotes the average time and S.D. represents the standard deviation across trials. Paths A and B refer to two consecutive pick-and-place tasks performed within each map. (N: Navigation, P1: Pick, and P2: Place).

☐			Path A				Path B				Total Time (s)
			N	P1	N	P2	N	P1	N	P2	
A	Baseline	A.T.	33.80	141.60	63.60	102.00	59.20	131.80	85.40	100.00	717
		S.D.	1.72	3.14	2.87	2.45	2.93	4.96	2.65	3.74	
	Ours	A.T.	34.80	138.00	60.00	97.00	54.60	127.20	83.40	98.40	693
		S.D.	2.56	1.90	2.37	2.45	1.72	1.83	3.14	2.50	
B	Baseline	A.T.	66.40	156.80	18.00	106.00	66.80	135.80	41.00	107.00	698
		S.D.	3.98	2.23	1.41	2.61	3.49	2.04	1.26	2.45	
	Ours	A.T.	65.60	153.80	16.00	101.00	61.60	130.60	38.00	103.00	670
		S.D.	3.32	2.14	1.10	2.00	3.01	2.50	1.41	2.10	
C	Baseline	A.T.	103.20	175.60	104.00	135.80	140.80	136.40	104.00	112.20	1012
		S.D.	2.64	5.08	4.05	4.07	3.87	3.93	3.22	3.97	
	Ours	A.T.	98.60	167.60	96.80	128.80	139.20	132.60	100.60	108.40	973
		S.D.	1.36	4.88	2.93	4.83	2.14	2.94	3.72	2.24	
D	Baseline	A.T.	35.20	136.20	74.80	185.20	64.80	250.60	38.60	100.40	886
		S.D.	2.48	1.72	2.99	2.48	2.64	3.26	2.06	3.38	
	Ours	A.T.	35.60	129.40	65.00	176.40	58.40	247.60	38.00	95.80	846
		S.D.	2.65	3.26	2.00	4.08	2.24	2.33	2.04	1.72	

### Appendix A.3

**Listing A2.** List of 100 common household objects used as examples in the prompt design to generate requests for moving items within a home environment.
{ “Objects”: [“remote control”, “book”, “phone”, “tablet”, “laptop”, “pillow”, “blanket”, “cup”, “glass”, “mug”, “plate”, “fork”, “spoon”, “knife”, “bottle “, “water bottle”, “vase”, “flower pot”, “toy”, “pen”, “pencil”, “notebook”, “scissors”, “hairbrush”, “comb”, “toothbrush”, “razor”, “soap”, “shampoo bottle”, “towel”, “key”, “wallet”, “bag”, “shoe”, “sock”, “hat”, “glove”, “ remote controller”, “controller”, “fan”, “vacuum cleaner”, “broom”, “dustpan “, “sponge”, “dish”, “pan”, “pot”, “spatula”, “whisk”, “knife sharpener”, “ cutting board”, “measuring cup”, “magazine”, “newspaper”, “candle”, “lighter “, “screwdriver”, “hammer”, “paintbrush”, “ruler”, “tape measure”, “stapler”, “eraser”, “charger”, “USB cable”, “headphones”, “earbuds”, “game console”, “ camera”, “thermostat remote”, “heater”, “humidifier”, “vacuum cleaner attachment”, “curtain rod”, “puzzle piece”, “picture frame”, “calendar”, “ coaster”, “tray”, “table cloth”, “napkin”, “paper towel roll”, “trash can lid “, “shopping bag”, “plastic container”, “lunchbox”, “tupperware”, “toolbox”, “clothes hanger”, “shoe box”, “umbrella”, “mirror”, “toilet paper roll”, “ plunger”, “hair dryer”, “toaster”, “kettle”, “iron”, “alarm clock”, “blue phone”] }

**Listing A3.** List of 100 flat surfaces commonly found in household environments and used as potential placement locations in prompt designs.
{ “Places”: [“kitchen counter”, “dining table”, “coffee table”, “bookshelf”, “nightstand”, “bedside table”, “cabinet”, “drawer”, “closet shelf”, “bathroom sink”, “kitchen sink”, “refrigerator shelf”, “freezer shelf”, “pantry”, “shoe rack”, “wardrobe”, “dresser top”, “desk”, “TV stand”, “fireplace mantel”, “windowsill”, “kitchen island”, “microwave oven”, “oven rack”, “stove top”, “dish rack”, “laundry basket”, “laundry room shelf”, “washing machine”, “drying rack”, “bathtub edge”, “shower shelf”, “toilet tank”, “medicine cabinet”, “mirror shelf”, “toilet paper holder”, “towel rack”, “hallway table”, “entryway bench”, “mudroom shelf”, “garage shelf”, “toolbox”, “work bench”, “storage bin”, “attic floor”, “basement shelf”, “under the bed”, “under the couch”, “sofa armrest”, “patio table”, “balcony shelf”, “garden shed”, “porch bench”, “poolside table”, “sideboard”, “hutch”, “china cabinet”, “wine rack”, “bar cart”, “cupboard”, “medicine drawer”, “fruit bowl”, “coat rack”, “hat stand”, “umbrella stand”, “shoe cabinet”, “recycling bin”, “trash can”, “compost bin”, “pet bed”, “pet food bowl”, “aquarium stand”, “fish tank”, “birdcage”, “dog crate”, “cat tree”, “window seat”, “bookshelf cubby”, “file cabinet”, “office desk drawer”, “printer stand”, “paper tray”, “pencil holder”, “mail organizer”, “kitchen towel rack”, “cooking utensil holder”, “cutlery drawer”, “spice rack”, “pantry shelf”, “refrigerator door”, “freezer drawer”, “under sink cabinet”, “cleaning supply shelf”, “vacuum cleaner storage”, “ironing board”, “coat closet”, “hat shelf”, “linen closet”, “towel closet”, “kids toy box”, “playroom shelf”, “craft room table”, “sewing machine stand”] }

**Listing A4.** Example of a JSON file generated using LLM-based data creation. The file includes fields such as request (a generated command for moving an object), object (the item to be moved), from (the current location of the object), destination (the target location of the object), and index (the data index). All fields, except for request, were used to verify whether our model accurately extracted and interpreted the relevant information, ensuring the robustness of the speech recognition module evaluation.
{ “Requests”: [ { “request”: “Please transfer the clothes hanger from the stove top to the entryway bench.”, “object”: “clothes hanger”, “from”: “stove top”, “destination”: “entryway bench”, “index”: 0 }, { “request”: “Could you put the blanket on the wine rack after taking it from the entryway bench?”, “object”: “blanket”, “from”: “entryway bench”, “destination”: “wine rack”, “index”: 1 }, { “request”: “Please place the pen on the sofa armrest from the birdcage.”, “object”: “pen”, “ from “: “birdcage”, “destination”: “sofa armrest”, “index”: 2 }, … { “request”: “Could you put the ironing board on the wine rack after taking it from the pet bed?”, “object”: “ironing board”, “from”: “pet bed“, “destination”: “wine rack”, “index”: 1899 } ] }
